# Storage Time in Bottle: Influence on Physicochemical and Phytochemical Characteristics of Wine Spirits Aged Using Traditional and Alternative Technologies

**DOI:** 10.3390/molecules30092018

**Published:** 2025-04-30

**Authors:** Sheila C. Oliveira-Alves, Tiago A. Fernandes, Sílvia Lourenço, Joana Granja-Soares, Andreia B. Silva, Maria Rosário Bronze, Sofia Catarino, Sara Canas

**Affiliations:** 1 INIAV—Instituto Nacional de Investigação Agrária e Veterinária, Quinta de Almoinha, Polo de Inovação de Dois Portos, 2565-191 Dois Portos, Portugal; silvia.lourenco@iniav.pt; 2 CEF—Centro de Estudos Florestais, Laboratório Associado TERRA, Instituto Superior de Agronomia, Universidade de Lisboa, Tapada da Ajuda, 1349-017 Lisboa, Portugal; 3 MINDlab—Molecular Design & Innovation Laboratory, Centro de Química Estrutural, Institute of Molecular Sciences, Departamento de Engenharia Química, Instituto Superior Técnico, Universidade de Lisboa, Av. Rovisco Pais, 1049-001 Lisbon, Portugal; tiago.a.fernandes@tecnico.ulisboa.pt; 4 DCeT—Departamento de Ciências e Tecnologia, Universidade Aberta, Rua da Escola Politécnica, 141-147, 1269-001 Lisboa, Portugal; 5 LEAF—Linking Landscape, Environment, Agriculture and Food Research Center, Instituto Superior de Agronomia, Universidade de Lisboa, Tapada da Ajuda, 1349-017 Lisboa, Portugalsofiacatarino@isa.ulisboa.pt (S.C.); 6 Associate Laboratory TERRA, Instituto Superior de Agronomia, Universidade de Lisboa, Tapada da Ajuda, 1349-017 Lisboa, Portugal; 7 iMed.ULisboa—Research Institute for Medicines, Faculdade de Farmácia, Universidade de Lisboa, Av. Gama Pinto, 1649-003 Lisboa, Portugalmrbronze@ff.ulisboa.pt (M.R.B.); 8 iBET—Instituto de Biologia Experimental e Tecnológica, Apartado 12, 2781-901 Oeiras, Portugal; 9 ITQB-NOVA—Instituto de Tecnologia Química e Biológica António Xavier, Universidade Nova de Lisboa, Av. da República, 2780-157 Oeiras, Portugal; 10 CEFEMA—Center of Physics and Engineering of Advanced Materials, Instituto Superior Técnico, Universidade de Lisboa, Av. Rovisco Pais, 1, 1049-001 Lisboa, Portugal; 11 MED—Mediterranean Institute for Agriculture, Environment and Development, Institute for Advanced Studies and Research & CHANGE—Global Change and Sustainability Institute, Universidade de Évora, Polo da Mitra, Ap. 94, 7006-554 Evora, Portugal

**Keywords:** spirit beverages, bottle storage, phenolic compounds, antioxidant activity, chestnut staves

## Abstract

Few studies have investigated the influence on physicochemical and phytochemical compositions during storage in the bottle of wine spirits (WSs) aged using alternative ageing technology (AAT) compared to traditional ageing technology (TAT). The aim of this study was to evaluate the effect of the bottle storage over one and four years on the evolution of chromatic characteristics (CIELab method) and physicochemical characteristics (alcoholic strength, acidity, and total dry extract), total phenolic index (TPI), low molecular weight compound contents (HPLC-DAD technique), in vitro antioxidant activities (DPPH, FRAP, and ABTS assays), and phenolic characterisation (HPLC-DAD-ESI-MS/MS technique) of WSs aged with chestnut wood using TAT (barrels, B) and AAT (micro-oxygenation levels (MOX): O15, O30, and O60; and control (N)). The results showed that after four years of storage in the bottle, the O60 modality resulted in smaller changes in physicochemical characteristics, higher preservation of phenolic content, and greater evolution of chromatic characteristics, ensuring its overall quality compared to other modalities. Antioxidant activity decreased similarly in both technologies, such as phenolic acid content, in particular, gallic acid content. According to the findings of this study, alternative ageing technology might be the best alternative for wine spirit quality and ageing process sustainability.

## 1. Introduction

The use of sustainable processes is fundamental for competitiveness within the beverage industry, contributing to its brand value, new market opportunities, and consumers’ preferences and purchase choices [1]. Integrating sustainability into industrial processes and operations contributes to addressing global challenges, such as climate change, pollution, energy consumption, and depletion of natural resources, contributing to the achievement of the Sustainable Development Goals (SDGs) of the United Nations [2,3].

In this scenario, one of the pillars of sustainability is the development of more eco-efficient processes, which will pave the way for the discovery and application of new technologies, as well as heightened awareness of environmental, economic, and social challenges [4,5]. As the majority of wine spirits (WSs) are produced in Europe, it is crucial to highlight the European plan for achieving sustainable production and ageing processes for spirit beverages by 2050 [6].

In particular, one of the strategies has been the use of sustainable wood-based ageing technology, an alternative ageing technology (AAT), which combines wood fragments (staves, chips, tablets, and cubes, among others) with micro-oxygenation (MOX) flow, applied to beverages stored in stainless steel tanks, aiming to mimic the ageing process that occurs in wooden barrels [7,8]. The ageing process of the wine distillate stands out for its significant contribution to the enrichment of the beverage in phenolic and volatile compounds, given the specific positive chemical and sensory characteristics conferred by the wood, depending on the botanical species [9,10,11,12].

From a legal point of view, in many countries that produce WSs, including Portugal, the legislation requires the use of “traditional practices” at this stage (traditional ageing technology—TAT), not allowing the use of AATs [13]. Given this sustainable approach, TAT has strong drawbacks, such as low production efficiency, high evaporation, high cost, longer ageing time, and high demand for wood, a natural resource with limited availability, contributing to a high environmental impact. For these reasons, the authors investigated the use of AAT for the ageing of WSs that combines wood staves with several micro-oxygenation (MOX) flows [7,14,15,16]. Several factors have encouraged the study using AAT, aiming strengthening the connection between sustainable practices and process optimisation in the production of aged WSs, which reduces time, cost, and environmental impact, while maintaining or even providing better quality [17]. In fact, from a long-term perspective, AAT does not aim to replace TAT. Rather, it aims provide the spirits industry with cost-effective and sustainable solutions, especially in situations of scarcity of natural resources, such as wood.

In previous studies, comparing sustainable ageing processes, the authors showed that AAT and TAT could be distinguished based on the concentrations of the volatile phenols, specifically on the higher concentrations of guaiacol, syringol, 4-methylguaiacol, 4-methylsyringol, and acetovanillone [14]. These compounds are highly appreciated by consumers, as syringol and 4-methylsyringol are associated with odour notes of wood and smoke, while guaiacol and 4-methylguaiacol confer toasted and burnt odours [9,14]. Likewise, higher total phenolic content and individual contents of low molecular weight phenolic compounds (syringic acid, ellagic acid, vanillin, syringaldehyde, coniferaldehyde, and sinapaldehyde) were obtained using AAT compared to TAT [7], contributing to a greater evolution of colour in WSs. Despite these positive results on some chemical and sensory features of the WSs during the ageing process using AAT, it is crucial to assess the overall quality, including phenolic content, colour, and physicochemical characteristics, of these aged WSs during their storage in the bottle.

Studies on the physicochemical and phytochemical compositions during storage in the bottle of WSs aged by traditional and alternative technologies (AAT) and comparison of both technologies are scarce. A previous study evaluated how the phenolic content changed over a year in bottled wine spirits aged with chestnut wood using AAT. It showed that the total phenolic content did not decrease, and the in vitro antioxidant activity was maintained [18]. During WS storage, oxygen present in bottle’s headspace and dissolved in aged WSs contributes to phenolic compounds undergoing oxidation and condensation reactions [18]. These compounds stay dissolved in the aged WS, evolving and causing changes in the organoleptic characteristics. Furthermore, during the oxidation process in the bottle, *o*-diphenols are oxidised to *o*-quinones and semi-quinone, and free radicals may be produced, while oxygen is reduced to H_2_O_2_ [19]. These cascade reactions and continuous modifications in the composition of the aged WSs in the bottle during storage can be affected by different parameters, such as temperature, oxygen content, light exposure, humidity, bottle position, time of storage, and types of closure [20].

The main objective of this study was to investigate whether the ageing characteristics obtained by the AAT are retained during storage in the bottle and to compare the WS characteristics obtained from both processes. This work is an extension of the investigation of the chemical evolution of aged WSs during storage in the bottle [18] of the Oxyrebrand Project (https://projects.iniav.pt/oxyrebrand/index.php/pt/, accessed on 14 January 2025), by studying the effect of three levels of MOX or nitrogen (control) applied to the same wine distillate kept in 50 L glass demijohns with chestnut wood staves inside (AAT) and compared to TAT (chestnut barrels) [15,16]. For the first time, the influence of bottle storage over four years, through a broader analytical approach, was determined, involving chromatic and physicochemical characteristics (alcoholic strength, acidity, and total dry extract), phenolic content (total phenolic index), low molecular weight compound concentrations (HPLC-DAD technique), in vitro antioxidant activities, and phenolic characterisation (HPLC-DAD-ESI-MS/MS technique) of the WSs aged with chestnut wood using TAT and AAT.

## 2. Results and Discussion

### 2.1. Effect of Storage Time in the Bottle on In Vitro Antioxidant Activities and Phenolic Content

**Figure 1:**
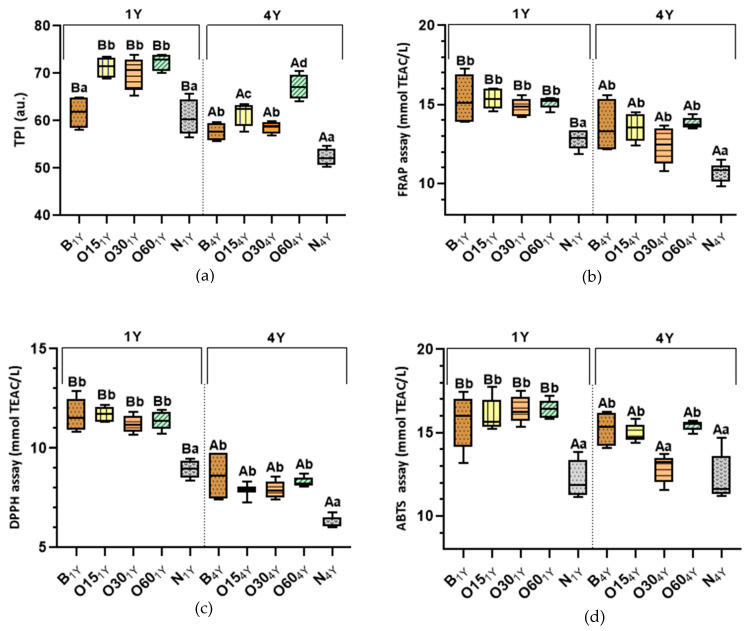
Box-plot diagrams of the average values of the total phenolic index (TPI) and antioxidant activities (AAs) of aged wine spirits over each storage time (one year (1Y) and four years (4Y)) according to ageing technologies (Traditional: B modality; Alternative: O15, O30, O60, and N modalities). Results are expressed as mean values ± standard deviation (*n* = 4). Phenolic determination: (**a**) TPI. AA determination: (**b**) FRAP assay; (**c**) DPPH assay; (**d**) ABTS assay. For each analytical determination, the different uppercase letters (A, B) in the box indicate significant differences between storage times (1Y and 4Y) for each ageing modality by the unpaired *t*-test (*p* < 0.05); and the different lowercase letters (a, b, c, d) in the box indicate significant differences between ageing modalities in each storage time by Tukey’s test (*p* < 0.05).

Previous research was conducted under the Oxyrebrand Project to enhance this alternative technology concerning WS quality and ageing sustainability, studying the evolution of phenolic compounds during storage in the bottle [18]. Phenolic compounds (PCs) present in aged WSs are predominantly extracted from the wood during the ageing process, being partly responsible for its aroma, taste, colour, and overall quality. Figure 1 shows the results concerning the determination of phenolic content (total phenolic index—TPI) and antioxidant activity (AA) of the aged WSs obtained using TAT (B modality) and AAT (O15, O30, O60, N modalities) after storage time in the bottle. During storage in the bottle, PCs undergo continuous oxidation and condensation reactions mediated by oxygen dissolved into aged WSs, which is also present in the headspace of the bottle, shifting and producing changes in the characteristics and quality of WSs [18,20,21]. A gradual evolution of spirit beverages occurs over storage in the bottle even in an environment with a very low oxygen content. Therefore, the determination of phenolic content throughout storage in the bottle is fundamental for understanding the ageing chemistry of WSs and determining whether the characteristics imparted by the ageing modality are preserved during their storage. Figure 1a shows the effect on TPI content of aged WSs after one and four years’ storage in the bottle.

The statistical analysis demonstrated that the TPI values of the alternative ageing technology (O15 (71.29 ± 2.19), O30 (70.11 ± 3.28), O60 (72.40 ± 1.71) were not significantly different from one another after bottle storage (1Y), but were significantly higher than those of the N modality (60.70 ± 3.51) and traditional technology (B modality (61.70 ± 3.22)). In 1Y, little difference in antioxidant activity values between the ageing modalities (O15, O30, and O60) may be ascribed to the MOX level and the wood variability (even with the same surface-to-volume ratio used). According to the experimental design of this study, the quantity of wood staves used in AAT was calculated to reproduce the surface area-to-volume ratio of traditional barrels. It is important to highlight that the surface area-to-volume ratio in AAT with MOX flows may not yield equivalent ageing effects due to the different nature of full stave contact [7,20,22]. In AAT, full stave contact combined with MOX flows (O15, O30, and O60 modalities) can promote more extraction, which affects the balance between additive and subtractive phenomena, resulting in higher phenolic content in WSs. These results agree with those reported by Anjos et al. [23] for WSs aged by Alternative Technology with chestnut staves.

After four years in the bottle (4Y), the results show that the TPI values of WSs from ageing modality O60 (67.21 ± 2.40) and B (57.66 ± 1.84) exhibited a similar reduction of approximately 7%, while O15 (61.54 ± 2.35), O30 (58.66 ± 1.27), and N (52.28 ± 1.65) displayed a reduction of approximately 14% compared to 1Y. Furthermore, the alternative ageing modalities (O15, O30, O60) had become significantly different from one another after bottle storage (4Y). Despite the reduction in TPI values in all modalities, the TPI value of the O60 modality was significantly higher than that of the other modalities (B, O15, O30, and N) and better retained the phenolic content. According to previous studies [24,25,26], the reduction in TPI values indicates the evolution of reactions that occur in alcoholic beverages during storage in the bottle. These reactions would presumably lead to changes in the antioxidant activity of aged WSs; therefore, this study assessed their AAs. It is crucial to highlight that there are competing mechanisms, such as gallic acid hydrolysis and galloyl-glucose oxidation. Gallic acid consumption shifts the hydrolysis balance, reducing galloyl-glucose ester production [27].

A global profile of the antioxidant activity of aged WSs can be evaluated using methods that assess hydrophilic and lipophilic antioxidant activities, which use single-electron transfer mechanisms (SET) [28]. SET-based assays, such as ABTS (2,2′-azino-bis(3-ethylbenzothiazoline-6-sulfonic acid), FRAP (ferric reducing antioxidant power), and DPPH (2,2-diphenyl-1-picrylhydrazyl), measure the capacity of an antioxidant in the reduction of an oxidant [29]. Studies show that ABTS, DPPH, and FRAP methods provide comparable values for the antioxidant activity in beverages [18,28]. Thus, the ABTS, DPPH, and FRAP methods were chosen to evaluate the AAs of aged WSs due to their solubility with the samples, straightforward operation, quick execution, and good repeatability and reproducibility.

After one year storage in the bottle (1Y), the antioxidant activities of the WSs aged by traditional (B modality (11.68; 15.33; 15.62 mmol TEAC/L)) and alternative technologies (O15 (11,71; 15.35; 16.03 mmol TEAC/L), O30 (11.19; 14.85; 16.35 mmol TEAC/L), and O60 (11.38; 15.11; 16.45 mmol TEAC/L) modalities) were not significantly different when measured by the DPPH, FRAP, and ABTS methods, respectively (Figure 1b–d). Except for the control modality (N: 8.96; 12.79; 12.21 mmol TEAC/L) from the alternative technology, where a significantly lower AA (DPPH, FRAP, and ABTS methods) was observed, while the other modalities behaved similarly. This resulted is expected, since lower phenolic content was obtained in this N modality (Figure 1a).

After four years of storage in the bottle (4Y), the N modality (10.72; 6.25; 12.27 mmol TEAC/L) maintained its profile, and its AA values were significantly lower compared to those of other modalities. Results show that the AA values of the ageing modalities (O15 (7.89; 13.56 mmol TEAC/L), O30 (7.93; 12.44 mmol TEAC/L), and O60 (8.31; 13.82 mmol TEAC/L)) were not significantly different when measured by the DPPH and FRAP methods, respectively. On the other hand, the ABTS method presented the lowest values for O30 (12.87 mmol TEAC/L) in relation the ageing modalities (B (15.25 mmol TEAC/L), O15 (14.91 mmol TEAC/L), O60 (15.44 mmol TEAC/L). According to the ABTS method, the radical ABTS˙ allows the determination of the antioxidant activity of both lipophilic and hydrophilic compounds [29]; therefore, a reduction of lipophilic compounds (such as fatty acids and aliphatic compounds) due to oxidation reactions in the O30 modality may explain this result. Furthermore, the O30 modality at 4Y presented different behaviour compared to the O15 and O60 modalities in all antioxidant assays, which was consistent with the reduction in its TPI value (Figure 1a). In fact, the antioxidant activity of phenolic compounds of aged WSs is not exclusively dependent on their overall quantity; but it also depends on the different polyphenolic structures formed during evolution in the bottle and their respective concentrations [18].

It is important to emphasise that the average results of FRAP and ABTS values after one year (1Y) were similar, with the exception of DPPH values, which were lower. This behaviour can be explained by the reaction mechanisms between antioxidants and radical DPPH˙, which depend on the structural conformation of the antioxidants [30,31,32]. Therefore, many antioxidants may react with different kinetics or may not react at all, estimating a lower antioxidant activity of the sample. However, according to Pearson’s correlation analysis of results, a positive and significant correlation was found between the values obtained TPI and AA methods (r∑_DPPH_ = 0.75; r∑_FRAP_ = 0.77; r∑_ABTS_ = 0.68) of all modalities, indicating the efficiency of these methods for the in vitro evaluation of antioxidant activity of aged WSs. Studies have demonstrated a positive correlation between phenolic content and AA for cognacs [33], brandies [34], and aged wine spirits [8,18].

Comparing all results, AA decreased significantly after four years of bottle storage compared to one year for both technologies, TAT and AAT (Figure 1b–d), displaying an average reduction of 28% in DPPH values, 13% in FRAP values, and 9% in ABTS values. PCs are unstable and highly prone to degradation and/or reaction with some factors, such as oxygen, light, and metal ions during their stages of storage, resulting in a change in the structures and a decrease in their antioxidant activity [19,20]. In fact, PCs are prone to auto-oxidation in the presence of oxygen, and peroxides and hydroperoxides are formed. Once auto-oxidation occurs, the concentration of PCs decreases, and oxidative polymerisation or degradation occurs, which is accompanied by a decrease in their bioactivity [35]. As expected, the O60 modality showed a lower reduction of AA values, which is consistent with TPI values (Figure 1a).

The results obtained suggest that a longer storage time results in a decrease in phenolic content and, consequently, in a reduction in the antioxidant activity of WSs aged in both technologies. These results are in accordance with those of Nowak et al. [36], who reported a decrease in antioxidant activity during long storage in the bottle (eight years) for fruit alcoholic beverage, due to a decrease in polyphenol content. A previous study [25] showed that herbal liqueur stored after one year in the bottle obtained a reduction of AA (DPPH assay) of 29%, while phenolic content reduction was 7%. Likewise, according to de Beer et al. [37], the antioxidant activities of red and white wines decreased after one year of storage in the bottles at 15 °C, displaying an average reduction of 17% to 21% (ABTS assay) and 29% to 59% (DPPH assay), respectively.

Several studies [8,35,37,38] confirm that the AA of aged WSs is dependent on their phenolic composition, which includes their chemical properties, oxidation degree, and concentration. In fact, each phenolic compound has an antioxidant activity profile based on its chemical structure and oxidation potential. Therefore, for our four year storage bottle samples, it was crucial to quantify and correlate LMW compounds with AA, in order to understand the contribution/correlation of these compounds to the AA values obtained. Table 1 depicts the concentrations of LMW compounds of each storage time (one and four years) according to the ageing modalities.

Based on these results, a positive Pearson’s correlation between AA values and total LMW compound concentration (r∑_DPPH_ = 0.82; r∑_FRAP_ = 0.71; r∑_ABTS_ = 0.61) was also observed, confirming that these compounds may play an important role in the antioxidant activity of aged WSs during storage time in the bottle (Table 1). In order to assess if a specific compound has more influence than others in determining the antioxidant activity, a correlation analysis was performed between the values of AA for each method and individual LMW compounds content of aged WSs. Strong Pearson’s correlations (0.99 to 0.50 indicate correlation) were observed only between antioxidant activity and LMW compound content for gallic acid (r∑_DPPH_ = 0.71; r∑_FRAP_ = 0.61; r∑_ABTS_ = 0.50) and sinapaldehyde (r∑_DPPH_ = 0.77; r∑_FRAP_ = 0.60; r∑_ABTS_ = 0.41), indicating that these compounds contribute to the overall antioxidant potential of the aged WS. The other phenolic acids and phenolic aldehydes (ferulic acid, vanillic acid, syringic acid, ellagic acid, vanillin, syringaldehyde, and coniferaldehyde) showed weak correlations with antioxidant capacity. Therefore, the antioxidant activity exhibited by aged WSs could be partially attributed to their richness in gallic acid (gall) and sinapaldehyde. These results are according to previous studies showing that gallic acid and sinapaldehyde act as antioxidants by scavenging the available free radicals, being able to reduce and inhibit the generation of free radicals [39,40,41,42].

Considering the results of the control modality (N) in each storage time in the bottle (1Y; 4Y), lower TPI and AA values were obtained, which is in line with the lower content of total LMW compounds (Table 1). These outcomes are in line with what was seen throughout the ageing experiment [15,16], which shows how important oxygen (MOX application) is for obtaining the target compounds out of AAT. On the other hand, the total LMW phenolic composition of the traditional technology was higher than the alternative technology, but the differences were not significant for the respective modalities (1Y: O30 and 4Y: O60). According to Table 1, the higher total LMW phenolic content for the traditional technology is directly related to its higher phenolic acid content. Total LMW concentrations for both technologies agree with the findings of previous studies [7,10]. During the ageing process [14,15], the MOX modalities (O15, O30, and O60) were designed to reproduce and accelerate reactions involving wood extractive compounds, such as those that occur in wooden barrels (B), but the results showed that these reactions were not similar, possibly due to differences in dissolved oxygen (DO) content. The dissolved oxygen content in the alternative technology (O15, O30, and O60) was higher (approximately 10 mg/L) than that observed in the wooden barrel (approximately 4 mg/L), given that the oxygen dissolved in a wooden barrel is always the result of a balance between the oxygen that passes through the wood and the oxygen that is consumed in the reactions [15,43].

Reduced levels of dissolved oxygen in the B modality compared to MOX modalities (O15, O30, and O60) may suggest increased consumption or a lower oxygen influx through the barrel, implying that this oxygen balance may play a role in attenuating the oxidative reactions of wood extractive compounds, resulting in a higher content of total phenolic acids in the B modality (Table 2). The application of micro-oxygenation in red and white wines has confirmed effects in the reduction of monomer and oligomeric phenolic compounds [44,45]. These results are in concordance with those obtained in previous research works [8,27,46], which reported higher concentrations of phenolic acids (gallic, ellagic, vanillic, and syringic acids) in aged WSs using the traditional technology in comparison with those acquired by the alternative technology (MOX combined with wood staves).

Regarding the evolution during storage in the bottle (4Y), the total LMW compounds reduced significantly in both technologies; however, for the O60 modality, this reduction was not significant (Table 1). It is worth highlighting that the TPI value of the O60 modality at 4Y was significantly higher than other modalities (B, O15, O30, N), which may explain this result, confirming better conservation of LMW compounds during bottle storage. The results obtained for the B modality (1Y) in this study are according to those found by Canas et al. [47], who reported that the content of total LMW compounds was 397.88 ± 37.07 mg/L. Also, the content of total LMW compounds was found to be the same using an alternative technology (320.00 ± 77.00 mg/L) [7]. Concerning total furanic aldehydes and total phenolic acids, a significant reduction was demonstrated in both technologies after four years of storage in the bottle (4Y), while for total phenolic aldehydes, the reduction occurred only in the traditional technology. The alternative technology better preserved their total phenolic aldehydes. Interestingly, phenolic aldehydes contribute positively to the aroma quality of aged WSs, imparting aroma characteristics such as cream, vanilla, cinnamon, spicy, and pepper, according to their thresholds [48].

Comparing each furanic aldehydes at 4Y, 5Mfurfural content reduced significantly for both technologies (overall 69%), while furfural and HMF contents exhibited similar behaviour, showing significant reductions for the B (11%; 21%), O30 (12%; 37%), and N (11%; 30%) modalities, respectively. Furfural oxidation may have occurred over time and can affect the aroma quality of aged WSs, as furanic aldehydes confer positive aromas, such as caramel, dry fruits and toasted almond [49,50]. Furfural oxidation is a simple oxidation reaction, in which a formyl group bonded to a furan ring is converted to carboxylic acid (5-methyl-2-furonic acid; furoic acid) [51]. Furthermore, furfuryl alcohol (2-furanmethanol) can be formed by degradation of furfuryl aldehydes during storage in the bottle [52].

Concerning phenolic aldehydes, a significant increase in syringaldehyde concentration was observed over time, while sinapaldehyde decreased significantly. These results can be explained by the oxidative cleavage of the double C-C bond of the aliphatic chain of these aldehydes, yielding the corresponding benzoic aldehydes; therefore, the oxidation of sinapaldehyde gives rise to syringaldehyde, which may be oxidised to syringic acid [10,53]. Vanillin and coniferaldehyde concentrations did not undergo a significant reduction. Vanillin, in particular, is one of the most important compounds contributing to a positive sensorial appreciation. This is because vanillin content is strongly associated with the intensity of vanilla and sweet aromas in wine spirits [48,49,54]. Furthermore, vanillin and syringaldehyde are commonly used as markers of the ageing process [55,56], and their preservation in aged WSs during bottle storage is a key factor in ensuring their quality and maintaining their market value.

The variations in phenolic acids, ferulic and syringic acid contents were not significant over time for both technologies. However, vanillic acid content increased significantly in the traditional technology, while it remained unchanged in the alternative technologies. The hydrolysis and oxidation of lignin-related compounds dissolved in aged WSs, such as 5-carboxyvanillic acid and *β*-hydroxypropiovanillone, contribute to the production of vanillic acid [57]. This may be one reason why the amount of this phenolic acid rises during storage.

In terms of ellagic acid content at 4Y, no significant changes were found for the B, O15, and O60 modalities, while the O30 and N modalities reduced by 13%. Ellagic acid is a condensed dimer of gallic acid [58]. Conversely, gallic acid content decreased for both technologies; the greatest reduction was observed for the alternative technology (overall 45%), followed by 20% for the traditional technology. Hernanz et al. [59] also demonstrated a reduction of 20% of gallic acid content after one year of storage in the bottle in white wine. Burin et al. [60] demonstrated a reduction of 50% of gallic acid content in red wines after 11 months of storage in the bottle. García-Falcón et al. [61] reported for red wines, after one year of bottle storage, an average reduction of 40% in gallic acid concentration, while vanillic acid and syringic acid concentrations did not change.

The reduction of gallic acid content may have occurred due to oxidation and dimerisation reactions. Gallic acid is susceptible to auto-oxidation in the presence of oxygen [62]. The three hydroxyl groups that are attached to gallic acid’s aromatic ring are prone to oxidation, which results in the formation of hydrogen peroxide, quinones, and semi-quinones [63,64]. Studies show that gallic acid oxidises and dimerises, regenerating the hydroquinone form (regenerative polymerisation), and then the dimer oxidises to give its quinone and hydrogen peroxide, consequently producing the oxidised dimer [65,66].

In general, phenolic oxidation occurs more slowly at low pH (characteristic pH of aged WSs), reduced oxygen concentration, and low temperature, which may explain the preservation of ferulic, vanillic, and syringic acid concentrations over time [63,64,67]. Furthermore, the antioxidant properties of phenolic acids are related to their redox potential and, therefore, a deeper knowledge of their oxidation–reduction behaviour is essential for a detailed understanding of the antioxidation process during bottle storage. Galato et al. [68] reported that gallic acid has a lower oxidation potential (Epa), higher redox potential (∆E), and higher antioxidant activity than vanillic and ferulic acids. In addition, the oxidation potential of ellagic acid is higher compared to gallic acid [69], which helps explain the relatively lower reduction of its content in aged WSs compared to gallic acid over time. Also, some research shows that gallic acid is a better antioxidant than ellagic and protocatechuic acids [70,71].

The free radical scavenging capacity of phenolic compounds is dependent on the number and position of hydroxyl groups, the presence of other functional groups, and mainly the ortho-hydroxyl arrangements in the aromatic ring. The antioxidant activity of a molecule increases with an increase in the number of hydroxyl groups attached to the aromatic ring [64,68]. Rice-Evans et al. [72] described that gallic acid has a total antioxidant activity of 3.0 mM relative to TEAC (Trolox Equivalent Activity Capacity), corresponding to the three available hydroxyl groups; however, its esterification in the carboxylate group decreases this capacity (2.4 mM). Equally, the substitution of the 3- and 5-hydroxyl with methoxy groups in syringic acid demonstrates an effective diminution in antioxidant activity (1.36 mM TEAC) compared to the trihydroxy derivative. Additionally, analysing furanic compounds by SET methods, HMF and 5Mfur did not show any antioxidant activity in DPPH and FRAP assays at concentrations below 200 mg/L [73]. Among phenolic aldehydes, their antioxidant activity decreased in this order: sinapaldehyde > syringic acid > coniferaldehyde [74]. Therefore, this depletion in antioxidant activity during long storage in the bottle is strongly correlated with the reduction of LMW compounds, specifically, sinapaldehyde and gallic acid.

In summary, oxygen concentration in the headspace of the bottle samples, as well as the previously dissolved oxygen in aged WSs, had a significant impact on the evolution of the phenolic compounds and antioxidant activity over time. The hydrolysis and oxidation reactions may be responsible for the changes in the phenolic composition of aged WSs during storage time in the bottle. Results showed that sinapaldehyde and gallic acid were the individual phenolic compounds that exhibited the most marked reductions after four years of storage in the bottle for both technologies. Among modalities, the O60 modality resulted in the highest preservation of phenolic content and antioxidant activity of aged WSs, ensuring its quality. According to this study, this technological alternative might be the most appropriate for wine spirit quality and ageing sustainability.

### 2.2. Effect of Storage Time in the Bottle on the Chromatic Characteristics of Aged WSs

Wooden barrels and staves used for the maturation of spirits are exposed to a heat treatment; as a result, toasted wood can release a greater number of LMW and Maillard reaction compounds (melanoidins, pyrazines, and furanic compounds) for distillate, which are partially responsible for the colour and aromas of aged WSs [75]. Colour is one of the fundamental attributes of an aged beverage’s appearance. Colour measurement of aged WSs has been used as an indirect measure of quality indicators, such as flavour, phenolic content, and physicochemical properties [16,76]. In fact, colour is the first quality parameter evaluated by consumers, contributing to impulse purchases; therefore, changes in the aged WS’s colour during long periods of storage in the bottle can impact its acceptance and market share.

The beverage industry has applied the CIELab method to measure and determine colour divergent from a set standard, as well as evolution during the storage time of the product. The CIELab method is based on the perception of just noticeable colour differences using the cylindrical coordinates of the system (L*, a*, b*). Thus, the determination of the chromatic characteristics L*, a*, and b* using the CIELab method allows to define the location of any colour in the uniform colour space [76,77]. The coordinate a* takes positive values for reddish colours, whereas b* takes positive values for yellowish colours [76]. In addition, L* is an approximate measurement of luminosity, taking values within the range 0–100 [78]. Chroma (C*) is a quantitative attribute of colourfulness and used to determine the degree of difference of a hue in comparison to a grey colour with the same Lightness. The higher the C* values of a sample, the higher is the colour intensity perceived by human eyes [76,78].

**Figure 2:**
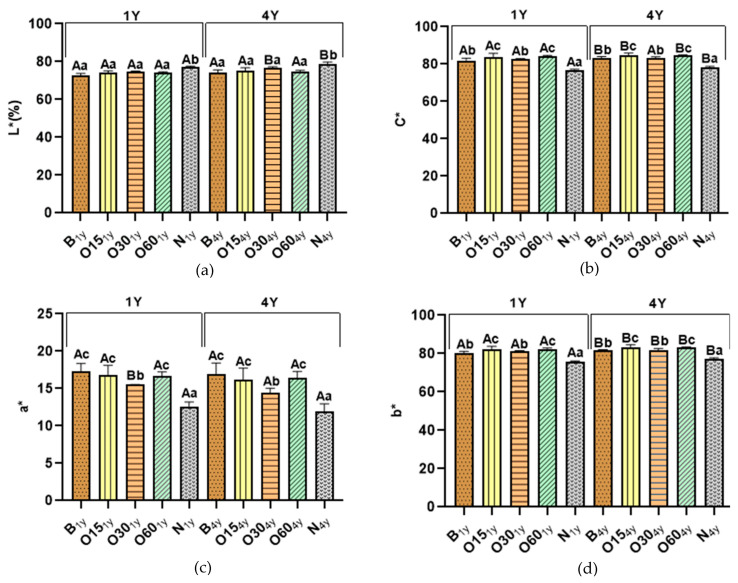
Effect of storage time in the bottle in the chromatic characteristics of wine spirits aged by ageing technologies (Traditional: B; Alternative: O15, O30, O60, and N): (**a**) Lightness (L*); (**b**) Chroma (C*); (**c**) chromaticity coordinate a*; (**d**) chromaticity coordinate b*. For each analytical determination, different uppercase letters (A, B) in the bars indicate significant differences between storage times (one year (1Y) and four years (4Y)) for each ageing modality by unpaired *t*-test (*p* < 0.05); and different lowercase letters (a, b, c, d) in the bars indicate significant differences between ageing modalities in each storage time by Tukey’s test (*p* < 0.05).

The chromatic characteristics of aged WSs after one and four years of storage in the bottle are shown in Figure 2.

Regarding each storage time (1Y and 4Y) in the bottle, the L* values of both technologies show no difference, except for the control (N) modality, as expected (Figure 2). Aged WSs from the N modality presented significantly higher L* values than the aged WSs obtained from other ageing modalities (B (72.85; 74.18), O15 (74.01; 75.38), O30 (74.93; 76.78); O60 (74.18; 74.93)), respectively (Figure 2a). This result is explained by the lower extraction of compounds from chestnut wood during the ageing process, which is confirmed by lower TPI values (Figure 1) and total LMW compound content (Table 1).

The statistical analysis demonstrated that after four years’ bottle storage, the L* values of the O30 (74.93; 76.78) and N (77.11; 78.68) modalities significantly increased, while no changes were observed in the B, O15, and O60 modalities. The higher L* values in the N and O30 modalities emphasise the role of oxygen during the ageing process and the oxidation of WS compounds during bottle conservation, as higher reduction of TPI values and total LMW compound content were observed in these modalities (Figure 1a and Table 1, respectively).

Concerning C* values (Figure 1b), a similar evolution/behaviour is observed for all ageing modalities for each storage time (1Y and 4Y) in the bottle, the O15 (83.85; 84.70) and O60 (84.09; 84.68) modalities obtained higher values compared to the B and O30 modalities, while the N modality (76.97; 78.98) showed significantly lower values. The B (81.97; 83.45) and O30 (82.52; 83.12) modalities did not differ significantly from each other. Interestingly, the B, O15, O60, and N modalities were significantly higher after four years’ bottle storage, while the O30 modality did not show any difference. The increase of C* values in all modalities confirms the presence of reaction mechanisms (such as oxidation, polymerisation, and hydrolysis, among others) during storage in the bottle, indicating the chemical evolution of aged WSs.

Figure 1c depicts the coordinate a* values of both technologies for each storage time (1Y and 4Y) in the bottle, demonstrating that B (17.33; 16.95), O15 (16.77; 16.20), and O60 (16.65; 16.41) displayed significantly higher values compared to the O30 (15,52; 14.47) and N (12.61; 11.94) modalities. In addition, the coordinate b* values (Figure 1d) exhibited the same behaviour of C* for all ageing modalities for each storage time (1Y and 4Y) in the bottle, O15 (82.15; 83.38) and O60 (82.42; 83.22) displayed higher values compared to B (80.11;81.70) and O30 (81.05; 81.85), while the N modality (75.92; 77.16) showed significantly lower values. It is important to highlight the significant increase of b* values (yellow hue) for both technologies after four years of storage in the bottle, while a* values were not significantly different, despite a slight downward trend. The increase of b* values contributed to higher values of C*.

In summary, aged WSs from the alternative technology, specifically the O15 and O60 modalities, demonstrated a greater colour evolution after four years of storage in the bottle due to the higher values of coordinate b* and C*, without significant changes in values of the coordinate a* and L*, confirming the role of oxygen in the development/evolution of colour. The oxidation of tannins and carboxyl ellagic acid in aged WSs responsible for the formation of compounds with yellow colour may explain the increase of coordinate b*, directly reflecting the increase of C* [79]. Furthermore, it is known that colour evolution during storage in the bottle of aged WSs can occur due to the oxidation and hydrolysis of phenolic compounds and furfural aldehydes, as well as other pigment degradations [18,22].

### 2.3. Effect of Storage Time in the Bottle on the Physicochemical Characteristics of Aged WSs

Physicochemical characteristics, mainly volatile, fixed, and total acidity are important parameters in determining high-quality spirits. Table 2 shows the effect of the storage time in the bottle on physicochemical characteristics of wine spirits aged by traditional and alternative technologies. It is worth noting that at each storage time (1Y and 4Y), the O15, O30, and O60 modalities presented significantly higher values of alcoholic strength by volume than the traditional technology (B modality). Because glass demijohns were used, the ethanol evaporation phenomenon of WSs was lower during the ageing process using the alternative technology. This is what was expected. After four years of storage in the bottle, the alcoholic strength values did not show significant changes in the aged WSs for both technologies. The closure and the permeability of the stopper (such as the effective diffusion coefficient) and the transfer of oxygen at the interface between the cork stopper and the glass bottleneck can contribute to the oxidation and evaporation of aged WSs during bottle ageing [80].

The statistical analysis demonstrated that the values of the total acidity, fixed acidity, and volatile acidity were significantly higher in the traditional technology than the alternative technology in each storage time (1Y and 4Y), which is characteristic of the ageing process in a barrel. A recent study demonstrated higher values of total acidity in WSs aged using traditional technology than alternative technology [46]. Among the modalities from the alternative technology, the O15 modality showed slightly higher values of total acidity, fixed acidity, and volatile acidity than the O30 and O60 modalities, while the control modality (N) was significantly lower. After four years of storage in the bottle, for the traditional technology, there was a reduction of total acidity and fixed acidity, while the volatile acidity was higher. Conversely, the values of total acidity and volatile acidity were significantly higher in the alternative technology, while for fixed acidity, no changes were observed.

During storage time in the bottle, volatile acidity was the factor contributing to the total acidity in aged WSs, resulting from the hydrolysis of acetyl groups to acetic acid [81,82]. Furthermore, the oxidation of acetaldehyde also may contribute to the acetic acid content of aged WSs [83]. The total acidity of aged WSs considers all types of acids, such as inorganic acids, organic acids, amino acids, and sulphurous acids, as well as phenolic acids. The increases of the total acidity can result from both the release of compounds from the hydrolysis of acetyl groups to acetic acid, and from the oxidation of aldehydes, esters, and alcohols to acids [61,84,85]. In addition, pH values were higher in the control (N) modality in each storage time (1Y and 4Y) compared to other modalities. Interestingly, pH values reduced in both technologies after four years of storage in the bottle. The pH value reflects the amount and strength of acids and is primarily dependent upon the total amount of existing acids in aged WSs [86]. The formation of acids resulting from the hydrolysis, oxidation, and condensation reactions of organic and phenolic compounds contributes to the decrease of pH value during bottle storage. A previous study reported the increase of total acids and decrease of total esters and pH value in Chinese distilled spirit (*Fenjiu*) after three years of storage in the bottle [87].

Regarding the total dry extract, there was no significant change for both technologies during storage time in the bottle, revealing a good conservation of the compounds resulting from the extraction/oxidation of wood that occurred during the ageing process. The total dry extract includes all matter that is non-volatile. Comparing the technologies, lower total dry extract values are observed only for the N modality in each storage time (1Y and 4Y), and the B, O15, O30, and O60 modalities did not display differences.

Indeed, the WSs aged alternative technology, specifically the O15, O30, and O60 modalities, showed similar behaviour in the changes on the physicochemical characteristics over four years of storage in the bottle compared to the B modality, indicating that different MOX levels weakly influence these parameters.

### 2.4. Multivariate Analysis

**Figure 3:**
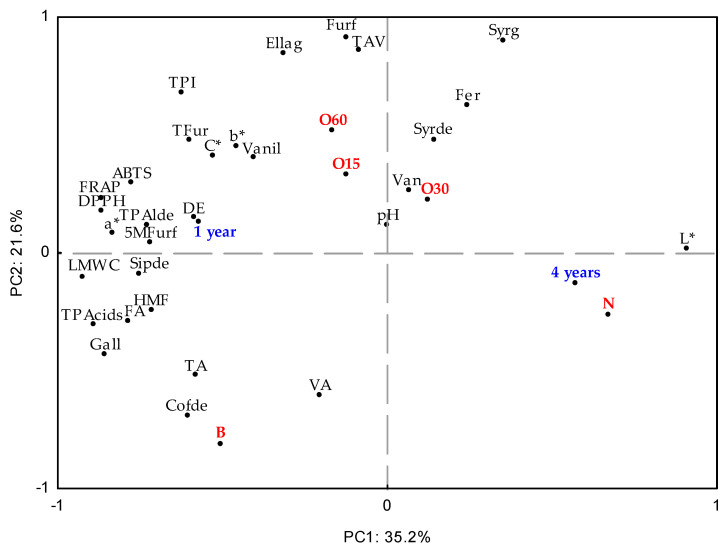
Principal Component Analysis (standardised scores and loadings) with results of chromatic characteristics (L*—Lightness, C*—Chroma, a*—chromaticity coordinate a*, b*—chromaticity coordinate b*); physicochemical parameters (TAV—alcoholic strength, TA—total acidity, FA—fixed acidity, VA—volatile acidity, DE—total dry extract, pH); antioxidant activity (DPPH, FRAP, ABTS methods); phenolic content (total phenolic index—TPI) and total and individual LMW (low molecular weight) compound content from aged WSs using ageing technologies (Traditional: B; Alternative: O15, O30, O60, and N) over storage time (one year (1Y) and four years (4Y)) in the bottles. LMW compounds: TLMWC—sum of content of individual LMW compounds; TPacids—sum of phenolic acids; TFur—sum of furanic aldehydes; TPalde—sum of phenolic aldehydes; Gall—gallic acid; Ellag—ellagic acid; Van—vanillic acid; Syrg—syringic acid; Fer—ferulic acid; Vanil—vanillin; Syrde—syringaldehyde; Cofde—coniferaldehyde; Sipde—sinapaldehyde; Furf—furfural; HMF—5-hydroxymethylfurfural; 5Mfurf—5-methyfurfural.

Principal Component Analysis (PCA) was used to investigate the effect on the aged WSs’ characteristics during storage in the bottle (Figure 3). At this stage, all data obtained were considered to assess similarities between the ageing modalities (antioxidant activities, TPI, LMW compound content, physicochemical, and chromatic determinations) during storage time in the bottle. The first and second components (PC1 × PC2) explained 56.8% of the total variability of results. PC1 (35.2%) was influenced by the storage time of the bottle, four years (4Y), phytochemical parameters, and the chemical composition of the WSs aged using both the alternative technology with micro-oxygenation (O15, O30, O60, and N modalities) and the traditional technology (B modality).

In addition, the O30 and N modalities are positively positioned in relation to PC1, while the O15 and O60 modalities are negatively positioned. The difference between the MOX modalities (O15, O30, and O60) and the control modality (N) was significantly demonstrated at the end of the ageing process, and it was maintained throughout bottle ageing [15,16]. In fact, the N modality presented a higher L* compared to other modalities (Figure 2). It can be noted that the O15 and O60 modalities show a more pronounced differentiation from the O30 modality according to the variables’ analytics. Regarding the variables analysed, there was separation along PC1: vanillic acid, syringic acid, ferulic acid, and syringaldehyde were positioned positively, while vanillin, ellagic acid, furfural, TPI, and antioxidant activity (ABTS, DPPH, and FRAP assays) were positioned negatively. These data agree with those described in Table 1 and Table 2. In addition, PC2 accounted for 21.6% of the variation associated with the traditional technology. Specifically, the B modality showed correlations with total LMW compound content and total phenolic acids.

In summary, these results show that the percentage of variation explained by PC1 and PC2 is low, indicating that there is weak differentiation of the aged WSs’ characteristics according to storage time in the bottle (one year and four years). When compared to other alternative technology modalities, the O60 modality resulted in smaller changes and higher retention of its phenolic content and chromatic characteristics, ensuring its overall quality.

### 2.5. Phenolic Characterisation of Four Years’ Bottle Storage

The LWM compounds from WSs aged by the traditional and alternative technologies were tentatively identified by the LC-DAD-ESI-MS/MS technique using an electrospray ionisation source (ESI) in both negative and positive ionisation modes. The ionisation conditions in the mass spectrometer were optimised to detect the m/z values corresponding to the precursor ions. Table 3 shows a list of 36 compounds tentatively identified, including their retention time (RT), UV absorption maximum (λmax), precursor ion, MS/MS product ions, identification of the ageing modality, and the bibliographic references to support the identification.

**Figure A1:**
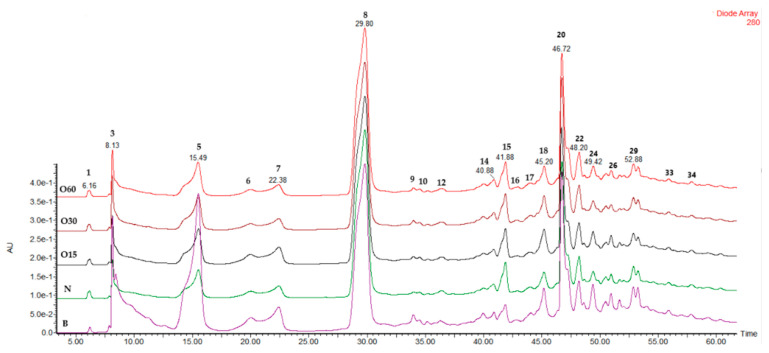
Chromatographic profile at 280 nm of wine spirits aged using ageing technologies (Traditional: B; Alternative: O15, O30, O60, and N) after four years of storage in the bottle.

The chromatographic profiles at 280 nm (Figure A1—Appendix A) of aged WSs from all modalities (B, O15, O30, O60, and N) were very similar, confirming that the alternative technology can mimic the traditional technology in terms of furanic aldehydes and phenolic profiles. A total of 36 LMW compounds were tentatively identified from all the modalities under study (Table 3): 2 organic acids (peaks 1 and 2), 2 furanic aldehydes (peaks 6 and 8), 13 phenolic acids (peaks 5, 7, 11, 12, 14, 15, 19, 21, 24, 25, 27, 29, and 36), 8 gallotannins (peaks 3, 4, 9, 10, 13, 16, 18, 20, and 28), 4 phenolic aldehydes (peaks 22, 23, 30, and 31), and 4 flavonoids (peaks 17, 25, 34, and 35). According to the literature, these phenolic acids, aldehydes phenolics, flavonoids, and hydrolysable tannins have been identified in heartwood extracts from chestnuts by the HPLC-DAD-ESI-MS/MS technique [95,103,104]. It is noteworthy that chestnut wood has been used in the ageing process of wine and spirit drinks due to its richness in hydroxybenzoic acids and hydrolysable tannins [95,106,107], as the presence of these compounds is confirmed in aged WSs (Table 3).

**Figure 4:**
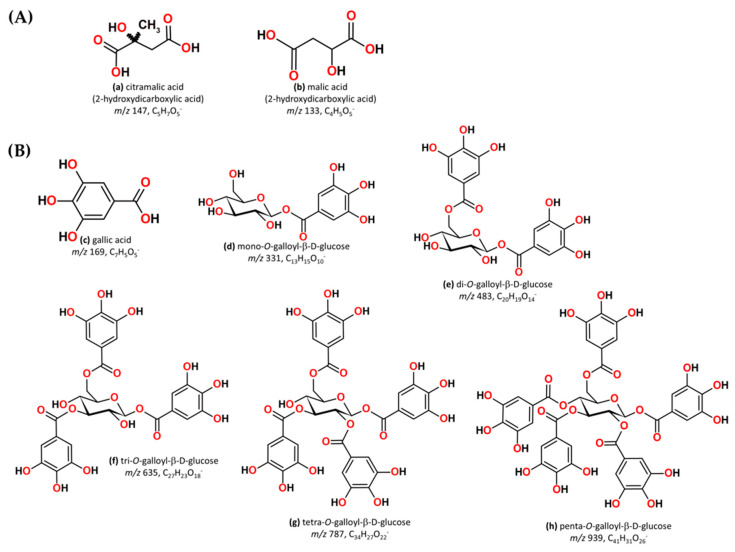
(**A**) Chemical structures of (**a**) citramalic acid and (**b**) malic acid; (**B**) Chemical structures of (**c**) gallic acid and gallotannins, (**d**) mono-*O*-galloyl-β-D-glucose, (**e**) di-*O*-galloyl-β-D-glucose, (**f**) tri-*O*-galloyl-β-D-glucose, (**g**) tetra-*O*-galloyl-β-D-glucose, and (**h**) penta-*O*-galloyl-β-D-glucose.

Organic acids, citramalic acid ([M − H]^−^ *m*/*z* 147), and malic acid ([M − H]^−^ *m*/*z* 133) were identified in aged WSs, originating directly from the grape and/or from the fermentation/distillation processes (Figure 4A). These organic acids play important roles in distilled alcohol beverages since they affect their organoleptic properties, stability, and acceptability [108]. Malic acid has been quantified in wine distillates, brandies, and whiskeys [108,109].

The precursor ions [M − H]^−^ at *m*/*z* 331, 483, 635, 787, and 939 are related to the gallotannin groups (Figure 4), belonging to a class of hydrolysable tannins [27]. Fernandes et al. [27] by a targeted metabolomics approach, depicted the loss of one or more galloyl groups (152 u) and/or gallic acid (170 u) from mono-, di-, tri-, and tetragalloyl glucopyranose, contributing to an increase in gallic and ellagic acid over time. In addition, isobaric gallotannins were highlighted, specifically di-O-galloyl-β-D-glucose with [M − H]^−^ at *m*/*z* 483 (peak 9 and 10). Studies have demonstrated that gallotannins exhibit a wide range of biological activities, including reduced incidence of cardiovascular disease, diabetes, cataracts, inflammation, and colorectal cancer, as well as the inhibition of tumour growth [110,111]. Furthermore, these compounds have astringency and antiradical properties, affecting the colour, roundness, and mouthfeel of aged WSs. In our previous study, we identified gallotannins in WSs aged with chestnut wood and explained their degradation pathway [27].

Notably, the esterification reactions of gallic acid may be confirmed by identification of digallate (peak 12) in aged WSs [27]. The oxidation–reduction reactions of flavonoids, on the other hand, were clearly observed in all modalities by identifying 3-carbethoxymethyl-flavone (peak 26), which showed the typical fragmentation pattern of this compound, with the product ions at *m*/*z* 261 and 179. The oxidation–reduction reactions of flavones to form methoxy-3,4-flavandiones and methoxy-3-(carbethoxymethylene) flavones has been demonstrated by Smith et al. [112]. Regarding flavonoids, Regalado et al. [94] identified 3-(carbethoxymethyl)-flavone and kaempferol in rum aged in oak barrels.

In general, phytochemical characterisation of aged WSs stored in the bottle for four years was performed, demonstrating that different groups of compounds, such as hydrolysable tannins, dimer phenolic acids, and oxidative flavonoids were maintained in a similar manner in both technologies, with these compounds being of crucial importance, because they are responsible for organoleptic characteristics and consumers’ acceptability.

## 3. Materials and Methods

### 3.1. Chemical and Reagents

Prior to the experiments, distilled water (conductivity < 6.0 µS/cm) and ultrapure water (conductivity < 0.055 µS/cm) were obtained from the Arium Comfort System (Sartorius, Goettingen, Germany). Acetonitrile (CH_3_CN, 99.9% *v*/*v*, LC gradient grade), methanol (MeOH, 99.9% *v*/*v*, LC gradient grade), and formic acid (HCOOH, 98% *v*/*v*, analytical grade) were purchased from Merck (Darmstadt, Germany), and ethanol (CH_3_CH_2_OH, 99.9% *v*/*v*, LC gradient grade) was purchased from Carlo Erba (Val de Reuil, France).

Antioxidant activities were performed using the DPPH (2,2-diphenyl-1-picrylhydrazyl) radical obtained from TCI (Tokyo, Japan), and TPTZ (2,4,6-tris(2-pyridyl)-s-triazine), ABTS (2,2′-azino-bis(3-ethylbenzothiazoline-6-sulfonic acid), Trolox (6-hydroxy-2,5,7,8-tetramethyl-chromane-2-carboxylic acid), and sodium acetate trihydrate purchased from Sigma-Aldrich (Steinheim, Germany). Iron (III) chloride hexahydrate (FeCl_3_·6H_2_O) and potassium persulfate (K_2_S_2_O_8_) were purchased from Honeywell Fluka (Seelze, Germany).

All the standard phenolic compounds (purity > 98%) were dissolved in ethanol/water (75:25, *v*/*v*) and stored in darkness at 7 °C before used. Ellagic acid dehydrate (ellag), vanillin (vanil), vanillic acid (van), syringic acid (syrg), ferulic acid (fer), 5-hydroxymethylfurfural (HMF), furfural (furf), and 5-methylfurfural (5mfurf) were purchased from Fluka (Buchs, Switzerland). Gallic acid (gall), 4-hydroxybenzaldehyde, syringaldehyde (syrde), coniferaldehyde (cofde), and sinapaldehyde (sipde) were bought from Sigma-Aldrich (Steinheim, Germany).

### 3.2. Experimental Design and Aged WS Sampling

**Figure 5:**
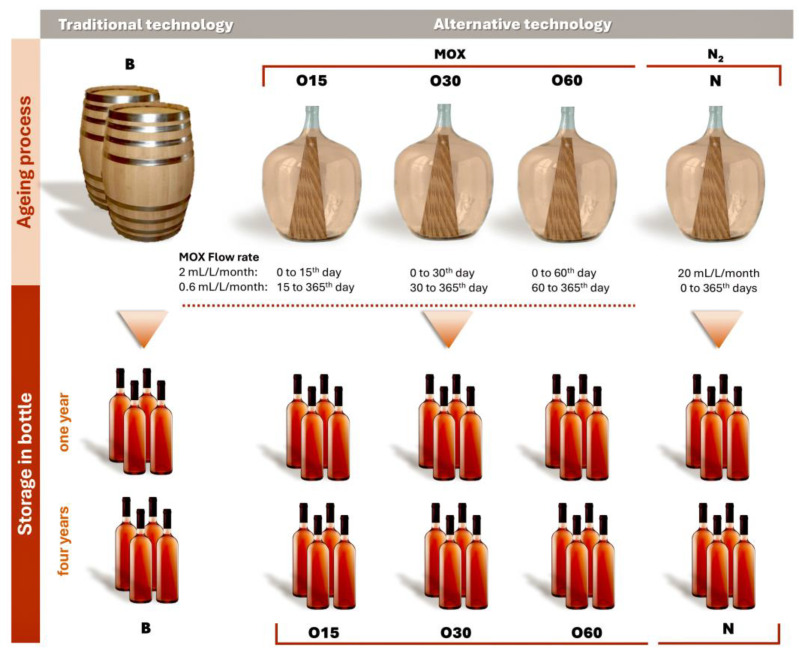
Illustration of the experimental design for the ageing trial of aged wine spirits with traditional and alternative ageing technologies.

This study involves an ongoing analysis of the chemical evolution of aged WSs during storage in the bottle [18] as part of the Oxyrebrand Project (https://projects.iniav.pt/oxyrebrand/index.php/pt/, accessed on 14 January 2025). The experimental design, consisting of two phases, was outlined in detail by Canas et al. [15,16] (Figure 5):

(1) The ageing trial was carried out on a pilot scale in 50 L glass demijohns, covering five ageing modalities: (i) chestnut barrels (B, representing the Traditional Ageing Technology) and (ii) three MOX modalities (O15, O30, O60) and one control modality with nitrogen (N) application (representing the alternative ageing technologies). Portuguese chestnut (*Castanea sativa* Mill.) barrels (250 L) and staves (50 cm length × 5 cm width × 1.8 cm thickness) were manufactured by J. M. Gonçalves cooperage (Palaçoulo, Portugal). The chestnut staves were toasted at a medium-plus toasting level (90 min at an average temperature of 240 °C; 1.8 cm of toasting thickness) in an industrial oven. In addition, the barrels were heated over a fire of wood offcuts under certain conditions of temperature to ensure a similar level of toasting. The quantity of staves inserted into the demijohns in four modalities (O15, O30, O60, N) was calculated to reproduce the surface area-to-volume ratio of a 250 L barrel (85 cm^2^/L). In this study, two replicates of each ageing modality were carried out. The wine distillate resulted from wine obtained from a mixture of several *Vitis vinifera* grape varieties cultivated in the Lourinhã Designation of Origin, including “Fernão-Pires”, “Alicante-Branco”, “Vital”, “Malvasia-Rei”, and “Cabinda”, from the 2018 harvest; the winemaking process involved free-run, followed by fermentation. The distillation process was conducted in a distillation column (Adega Cooperativa da Lourinhã, Lourinhã Designation of Origin, Lourinhã, Portugal). The wine distillate (alcoholic strength by volume, 78.3% *v*/*v*; total acidity, 0.12 g acetic acid/L of absolute ethanol; volatile acidity, 0.09 g acetic acid/L of absolute ethanol; pH, 5.33) was used to fill the barrels and demijohns.

MOX was applied to the WS during the ageing time, with pure oxygen (X50S Food, Gasin, Portugal) supplied through a multiple diffuser micro-oxygenator (VISIO 6, Vivelys, France) with ceramic diffusers, at different flow rates according to the ageing modality (O): (i) O15—50 L glass demijohns with chestnut staves and micro-oxygenation (flow rate of 2 mL/L/month during the first 15 days followed by 0.6 mL/L/month until 365 days); (ii) O30—50 L glass demijohns with chestnut staves and micro-oxygenation (flow rate of 2 mL/L/month during the first 30 days followed by 0.6 mL/L/month until 365 days); and (iii) O60—50 L glass demijohns with chestnut staves and micro-oxygenation (flow rate of 2 mL/L/month during the first 60 days followed by 0.6 mL/L/month until 365 days).

Pure nitrogen (X50S Food, Gasin, Portugal) was applied in the control modality (N) continuously (flow rate of 20 mL/L/month) over the ageing time through a specific device (Gasin, Portugal). The N modality aims to minimise dissolved oxygen in WSs, thus acting as a control. During the ageing process, the ten experimental units were stored in the cellar of Adega Cooperativa da Lourinhã (Lourinhã, Portugal) in the same environmental conditions.

(2) Storage in the bottle: after 365 days of the ageing process, the ten aged WSs were bottled on the same day in amber glass bottles (750 mL, two bottles from each demijohn), ensuring the same level of WS in each bottle. Regarding the bottle, the headspace was set at 9.8 mL of air in all bottles to ensure that the oxygen ingress into the aged WSs was similar, thereby controlling oxidation and allowing the main effects observed to be attributable to the ageing modality and storage time [80]. The cork stoppers were sealed with parafilm (Parafilm^®^, Bemis Company, Neenah, WI, EUA) to reduce evaporation. The bottles were transported in the same day and stored in the cellar of INIAV—Dois Portos at 19 °C and 80% relative humidity for 48 months. Sampling was carried out at one and four years after bottling. These sampling times were selected to carry out physicochemical and phytochemical analyses, based on two reasons: (i) this work is a continuity of a previous study performed under the Oxyrebrand Project, as aforementioned, in which the chemical evolution of aged WSs over 12 months of storage in the bottle was assessed; (ii) according to our team’s knowledge, for this spirit beverage, a storage period of at least one year in the bottle is usual to promote the desired balance. Long-term bottle storage in the cellar is common and also valuable (two to eight years), so four years was chosen as an average time. The data set included two technical replicates of each ageing modality (B, O15, O30, O60, and N)—Figure 5. Thus, a total of 40 samples [2 replicates of each modality (5 modalities) × 2 sampling bottles of each replicate × 2 storage times (one and four years)] of WSs were taken and analysed to determine the chromatic and chemical characteristics, total phenolic index, antioxidant activities, low molecular weight composition, phenolic profile, and their correlations as well.

### 3.3. Chromatic Characteristics

The chromatic characteristics (Lightness (L*), Chroma (C), chromaticity coordinates (a* and b*), and absorbance at 470 nm) of aged WSs were determined by the CIELab/CIELCh method [76] using a Varian Cary 100 Bio spectrophotometer (Santa Clara, CA, USA) and a 10 mm glass cell, as described by Canas et al. [7]. Transmittance measurement was carried out every 10 nm from 380 to 770 nm, using a D65 illuminant and a 10◦ standard observer. The analyses were performed in triplicate.

### 3.4. Physicochemical Characteristics

The basic chemical characteristics of the aged WSs were determined in duplicate: alcoholic strength by volume, pH, total dry extract, total acidity, fixed acidity, and volatile acidity. Alcoholic strength was obtained by distillation and electronic densimeter (DMA 5001, Anton Paar, Graz, Austria) [113]; the corresponding results were expressed as a volumetric percentage of ethanol in the WS. Total dry extract was analysed by gravimetry [35]; the corresponding results were expressed as grams per litre. pH was determined by potentiometry [35] using a potentiometer (micro pH2002, Crison, Barcelona, Spain) with a glass electrode and reference electrolyte (lithium chloride (LiCl) in an ethanol medium (1 mol/L) [113]. Total acidity was assessed by colorimetric titration and fixed acidity by colorimetric titration of the water solution of dry extract [81], and the corresponding results were expressed as grams of acetic acid per litre of absolute ethanol. Volatile acidity was obtained by calculation of the total acidity minus fixed acidity [81]. The analyses were performed in triplicate for each bottle. The total phenolic index (TPI) of the aged WSs was determined according to Cetó et al. [114]. Briefly, aged WSs were diluted with distilled water (1:100, *v*/*v*), and the absorbance was measured directly at 280 nm using a Varian Cary 100 Bio spectrophotometer (Santa Clara, CA, USA) and a 10 mm quartz cuvette. The TPI value of each bottle was calculated by multiplying the measured absorbance by the dilution factor. The analyses were performed in triplicate.

### 3.5. In Vitro Antioxidant Activity Analyses

#### 3.5.1. ABTS Assay

The ABTS assay was obtained according to the method reported by Rufino et al. [115], with some modifications. Briefly, the ABTS^•+^ radical cations were prepared by reacting 4 mL of a 7 mmol/L ABTS stock solution with 70.4 μL of 140 mmol/L potassium persulfate solution for 16 h, at room temperature and in the dark. Thereafter, the ABTS solution was diluted by adding ethanol (99.5%) to the ABTS^•+^ radical solution until the measured absorbance reached 0.700 ± 0.02 at 734 nm using a Varian Cary 100 Bio spectrophotometer (Santa Clara, CA, USA). The aged WSs were diluted with absolute ethanol (1:50 *v*/*v*). In a tube, 3 mL of ABTS solution was added to 30 μL of the sample or 30 μL of the standard solution, mixed, and placed in a water bath (Selecta Digiterm 3000542, Barcelona, Spain) for 6 min at 30 °C. After reaction, the absorbance was measured at 734 nm at room temperature. The control absorbance (30 µL of absolute ethanol plus 3 mL of ABTS solution) was measured at the beginning and end of the assay. Trolox standard curve (0.08–2.0 mM TEAC) was used as a reference antioxidant. Results were expressed as mmol Trolox equivalent antioxidant capacity (TEAC)/L of WS. The analysis was carried out in triplicate.

#### 3.5.2. DPPH Assay

The DPPH (2,2-diphenyl-1-picrylhydrazyl) free radical scavenging activity was carried out using the method described by Nocera et al. [8]. Briefly, 10 µL of aged WS was added to 3 mL of 8.5 × 10^−5^ M DPPH methanolic solution in a glass tube wrapped with aluminium foil. The tube was vortexed for 10 s and then immediately placed in a water bath (Selecta, Digiterm 3000542, Barcelona, Spain) at 30 ± 1 °C for 60 min; the tube was shaken every 10 min and placed back in the water bath. After cooling to room temperature, the absorbance was measured at a wavelength of 515 nm using a Varian Cary 100 Bio spectrophotometer (Santa Clara, CA, USA). The control absorbance (10 µL of methanol and 3 mL of 8.5 × 10^−5^ M DPPH methanolic solution) was measured at the beginning and end of the assay. Trolox was used as a reference standard curve (1–15 mM TEAC), and the results were expressed as mmol Trolox equivalent antioxidant capacity (TEAC)/L of WS. The analyses were performed in triplicate.

#### 3.5.3. FRAP Assay

The ferric reducing ability was determined by the FRAP (Ferric Reducing Antioxidant Power) method, according to Benzie and Strain [116] and Pulido et al. [117] with modifications. Briefly, the FRAP reagent was prepared with 75 mL of a 0.3 M sodium acetate buffer (pH 3.6), 7.5 mL of 10 mmol/L TPTZ (2,4,6-tris(2-pyridyl)-s-triazine) in a 40 mmol/L HCl solution, and 7.5 mL of 20 mmol/L FeCl_3_·6H_2_O in the dark. The aged WSs were diluted with ethanol (1:50, *v*/*v*). Sample or standard solutions, distilled water, and the FRAP reagent (90 µL sample or standard solution, 270 µL distilled water, and 2.7 mL FRAP reagent) were mixed and kept in a water bath (Selecta, Digiterm 3000542, Barcelona, Spain) for 30 min at 37 °C. After cooling to room temperature, absorbance was measured at 595 nm using a Varian Cary 100 Bio spectrophotometer (Santa Clara, CA, USA). The control absorbance (90 µL ethanol, 270 µL distilled water, plus 2.7 mL FRAP reagent) was measured at the beginning of the assay. A Trolox standard curve was also prepared (0.08–1.5 mM TEAC). Results were expressed as mmol Trolox equivalent antioxidant capacity (TEAC)/L of WS. The assay was performed in triplicate.

### 3.6. Analyses of Low Molecular Weight Compounds

#### 3.6.1. HPLC-DAD-ESI-MS/MS Identification

The characterisation of the phenolic compounds of aged WSs was performed in a Waters Alliance 2695 HPLC system (Waters, Milford, MA, USA) equipped with a quaternary pump, solvent degasser, autosampler, and column oven, coupled to a diode array detector (Detector Waters 2996, Milford, MA, USA). For the separation of compounds, a reversed-phase C18 column (LiCrospher 100 RP-18, 250 × 4 mm; 5 µm) in a thermostatic oven at 35 °C was used. A pre-column (100 RP-18, 5 µm) was also used. The mobile phase consisted of water/formic acid (99.5%/0.5%) as eluent A and acetonitrile/formic acid (99.5%:0.5%) as eluent B at a flow rate of 0.30 mL/min. All solvents were filtered through a 0.22 mm PVDF membrane (Millipore, Billerica, MA, USA) prior to analysis. The system was run with the following gradient elution program: 0–10 min from 99 to 95% A; 10–30 min from 95 to 82% A; 30–44 min from 82 to 64% A; 44–64 min at 64% A; 64–90 min from 64 to 10% A; 90–100 min at 10% A; 100–101 min from 10 to 95% A; 101–120 min at 95% A; and a final step to return to the initial conditions. The injection volume was 20 µL. DAD was used to scan the wavelength absorption from 200 to 650 nm. Tandem mass spectrometry (MS/MS) detection was performed using an electrospray ionisation source (ESI) at 120 °C, applying a capillary voltage of 2.5 kV, cone voltage of 30 V, and collision energy of 20 eV. The compounds were ionised in the negative and positive modes, and spectra were recorded in the range of *m*/*z* 60–1500. Ultra-high-purity argon (Ar) was used as a collision gas. High-purity nitrogen (N_2_) was used both as a drying gas and nebulising gas. For data acquisition and processing, MassLynx software (version 4.1, Waters Corporation, Milford, MA, USA) was used to control the analytical conditions and to collect the data from HPLC-DAD-MS/MS. For compound identification purposes, mass and UV spectra were compared with spectra already published in the literature. When standards were commercially available, the identification was based on the comparison of their fragmentation patterns and retention times.

#### 3.6.2. Low Molecular Weight Composition Determination

Low molecular weight (LMW) compounds of aged WSs were quantified according to the method of Canas et al. [118]. Chromatography separation of compounds was performed using an HPLC Lachrom Merck Hitachi system (Merck, Darmstadt, Germany) equipped with a quaternary pump L-7100, a column oven L-7350, a UV-Vis detector L-7400, and an autosampler L-7250, coupled with HSM D-7000 software (Merck, Darmstadt, Germany) for the management, acquisition, and treatment of data. A LiChrospher RP 18 (5 μm, 250 mm × 4 mm ID) column (Merck, Darmstadt, Germany) was used as a stationary phase. The mobile phase consisted of water/formic acid (98:2 *v*/*v*) as eluent A and methanol/water/formic acid (70:28:2 *v*/*v*/*v*) as eluent B, at a flow rate of 1 mL/min and column temperature of 40 °C. All solvents were filtered through a 0.45 µm PVDF membrane (Cronus filter, Gloucester, UK) prior to analysis. The autosampler’s temperature was set at 18 °C, and the injection volume was 20 µL. Samples were spiked with an internal standard (20 mg/L of 4-hydroxybenzaldehyde). The elution program was as follows: 0–3 min at 0% isocratic B; 3–25 min from 0% to 40% B; 25–43 min from 40% to 60% B; 43–55 min at 60% isocratic B; 55–60 min from 60 to 80% A; 60–65 min at 80% isocratic B; 65–75 min from 80 to 0% B; and finally returning to the initial conditions. Detection was made at 280 nm for phenolic acids (gall, van, syrg, fer, and ellag acids) and furanic aldehydes (HMF, furf, and 5mfurf) and at 320 nm for phenolic aldehydes (vanil, syrde, cofde, and sipde). Quantification of these compounds was performed through calibration curves (mg/L).

### 3.7. Statistical Analysis

The data of antioxidant activities, TPI, low molecular weight composition, and physicochemical parameters were expressed as mean ± standard deviation of the technical replicates of each ageing modality (B, O15, O30, O60, and N). One-way analysis of variance (ANOVA) was applied to assess the effects of the ageing modalities on the antioxidant activities, TPI, and LMW compound contents of the aged WSs for each storage time in the bottle. Another one-way ANOVA was carried out to assess the significance of the antioxidant activities, TPI, and LMW compounds over storage time in the bottle. Tukey’s test was performed to compare the average values when a significant difference (*p* < 0.05) was found. The correlation between antioxidant activities, TPI, and LMW compound concentrations were determined through Pearson’s correlation coefficient test, considering a confidence level of 95% (*p* < 0.05). Principal component analysis (PCA) of results was used to evaluate the possible grouping of chromatic characteristics, physicochemical parameters, TPI, and total LMW compounds for all modalities during storage time in the bottle. Statistical analysis and PCA were performed using Statistica version 7.0 (StatSoft Inc., Tulsa, OK, EUA). The results of the scores and loadings were standardised and presented in the same graphic, identifying the influence of each factor.

## 4. Conclusions

The findings presented herein enhance the understanding of the impact on the physicochemical and phytochemical characteristics of WSs aged with chestnut wood using traditional and alternative technologies over four years of storage in the bottle. During bottle ageing, the O60 modality enabled greater preservation of the phenolic content, demonstrating a higher total phenolic index value and a lower reduction of the phenolic acids and phenolic aldehydes in relation to the other modalities. The antioxidant activity values and phytochemical characterisation of aged WSs from traditional and alternative technologies showed comparable profiles. Positive correlations were found for total phenolic content and antioxidant activity; in particular, gallic acid content showed the highest correlation, followed by sinapaldehyde content. The experimental conditions allowed the identification of gallic acid derivatives, such as gallic acid esters and gallotannins, in all modalities. The results demonstrated an increase in b* coordinates and Chroma values in all modalities, evidencing colour evolution through the formation of yellowish compounds. In conclusion, few changes were noted in the alternative technology via the O60 modality after four years of storage in the bottle, suggesting that this eco-efficient ageing technique appears to preserve the overall quality of wine spirits. This study supplies new insights into the evolution of phenolic compounds of aged wine spirits during storage time in the bottle using alternative technology, potentially providing for spirits industry a cost-effective and sustainable solution.

## Figures and Tables

**Table 1:** Evolution of LMW compounds from aged wine spirits after one year and four years of storage in the bottle.

LMW Compounds (mg/L)	1Y					4Y				
	B_1Y_	O15_1Y_	O30_1Y_	O60_1Y_	N_1Y_	B_4Y_	O15_4Y_	O30_4Y_	O60_4Y_	N_4Y_
5-Hydroxy- methylfurfural	40.75 ± 1.46 Ba	33.38 ± 11.36 Aa	30.41 ± 4.68 Ba	26.63 ± 1.63 Aa	29.72 ± 5.80 Ba	31.89 ± 0.73 Ab	20.94 ± 6.72 Ab	18.91 ± 2.71 Aa	20.90 ± 7.48 Ab	17.35 ± 4.21 Aa
Furfural	51.92 ± 1.72 Ba	72.81 ± 5.83 Ac	67.94 ± 3.73 Bc	72.42 ± 3.55 Ac	62.95 ± 0.97 Bb	46.49 ± 1.80 Aa	64.83 ± 5.80 Ac	59.39 ± 2.88 Ac	67.16 ± 4.19 Ac	56.16 ± 0.99 Ab
5-Methylfurfural	0.87 ± 0.20 Bb	0.82 ± 0.23 Bab	0.71 ± 0.09 Bab	0.60 ± 0.15 Bab	0.48 ± 0.17 Ba	0.27 ± 0.11 Aa	0.21 ± 0.090 Aa	0.21 ± 0.12 Aa	0.34 ± 0.04 Aa	0.19 ± 0.06 Aa
Gallic acid	171.70 ± 12.74 Bc	122.50 ± 14.73 Bb	102.30 ± 14.41 Bab	104.60 ± 1.08 Bab	84.14 ± 15.17 Ba	137.70 ± 17.51 Ab	58.06 ± 7.26 Aa	59.36 ± 8.60 Aa	67.34 ± 18.52 Aa	41.66 ± 8.34 Aa
Ellagic acid	17.72 ± 1.03 Aa	21.73 ± 2.29 Abc	21.83 ± 0.86 Bbc	23.44 ± 0.45 Ac	19.31 ± 0.90 Bab	16.17 ± 0.71 Aa	18.68 ± 1.73 Aa	19.04 ± 0.89 Aa	22.91 ± 2.84 Ab	16.72 ± 0.55 Aa
Vanillic acid	12.58 ± 0.40 Aa	17.42 ± 5.83 Aa	17.18 ± 2.41 Aa	15.01 ± 0.14 Aa	16.07 ± 2.59 Aa	16.62 ± 2.86 Ba	16.29 ± 6.25 Aa	17.78 ± 3.91 Aa	15.05 ± 0.94 Aa	15.15 ± 3.48 Aa
Syringic acid	6.02 ± 0.50 Aa	13.12 ± 1.49 Ab	12.16 ± 0.92 Ab	12.97 ± 0.85 Ab	11.27 ± 0.72 Ab	6.75 ± 0.26 Aa	13.51 ± 0.99 Ac	13.10 ± 0.76 Abc	14.31 ± 1.12 Ac	11.48 ± 1.03 Ab
Ferulic acid	0.26 ± 0.03 Aa	0.37 ± 0.11 Aab	0.46 ± 0.08 Ab	0.43 ± 0.04 Ab	0.33 ± 0.02 Aab	0.29 ± 0.04 Aa	0.55 ± 0.15 Ab	0.50 ± 0.13 Aab	0.48 ± 0.12 Aab	0.37 ± 0.03 Aab
Vanillin	6.17 ± 0.08 Ab	6.53 ± 0.20 Abc	6.29 ± 0.27 Abc	6.88 ± 0.29 Ac	4.94 ± 0.49 Aa	6.50 ± 0.35 Aab	6.51 ± 0.46 Aab	6.32 ± 0.12 Aab	7.93 ± 1.99 Ab	5.15 ± 0.56 Aa
Syringaldehyde	13.59 ± 0.19 Aa	17.20 ± 0.11 Ab	16.01 ± 0.49 Ab	17.50 ± 0.38 Ab	13.53 ± 1.89 Aa	17.59 ± 0.78 Ba	20.99 ± 0.91 Bab	19.82 ± 0.88 Bab	24.30 ± 5.43 Bb	16.68 ± 2.30 Ba
Coniferaldehyde	9.17 ± 0.35 Ab	5.82 ± 0.23 Aa	5.81 ± 0.31 Aa	6.09 ± 0.89 Aa	5.62 ± 0.27 Aa	8.90 ± 0.23 Ab	5.57 ± 0.54 Aa	5.51 ± 0.37 Aa	5.96 ± 0.88 Aa	5.36 ± 0.05 Aa
Sinapaldehyde	30.36 ± 1.43 Bb	25.45 ± 0.39 Bba	23.98 ± 0.85 Ba	27.06 ± 2.25 Ba	24.96 ± 1.40 Ba	21.47 ± 2.29 Aa	17.04 ± 2.91 Aa	15.95 ± 2.95 Aa	20.51 ± 2.87 Aa	17.43 ± 1.15 Aa
Total furanic aldehydes	93.54 ± 3.27 Ba	107.01 ± 16.91 Ba	99.06 ± 1.57 Ba	99.65 ± 5.32 Ba	93.16 ± 6.25 Ba	78.65 ± 2.49 Aa	85.97 ± 12.62 Aa	78.51 ± 1.39 Aa	88.40 ± 11.54 Aa	73.69 ± 4.82 Aa
Total phenolic acids	208.30 ± 12.83 Bc	175.20 ± 5.76 Bb	153.90 ± 16.91 Bab	156.40 ± 1.13 Bab	131.10 ± 17.17 Ba	177.50 ± 21.18 Ab	107.10 ± 6.02 Aa	109.80 ± 12.80 Aa	120.10 ± 19.39 Aa	85.37 ± 10.61 Aa
Total phenolic aldehydes	59.28 ± 1.91 Bb	54.99 ± 0.57 Ab	52.09 ± 1.71 Aab	57.53 ± 3.75 Ab	49.04 ± 3.92 Aa	54.46 ± 1.68 Ab	50.10 ± 3.83 Aab	47.60 ± 3.55 Aab	58.69 ± 10.81 Ab	44.62 ± 2.36 Aa
Total LMWC	361.10 ± 12.85 Bc	337.10 ± 12.24 Bbc	305.10 ± 17.44 Bb	313.60 ± 7.91 Ab	273.30 ± 20.67 Ba	310.60 ± 24.48 Ac	243.20 ± 17.76 Aab	235.90 ± 14.98 Aab	267.20 ± 40.99 Abc	203.70 ± 12.64 Aa

Results are expressed as mean values ± standard deviation (*n* = 4) of aged wine spirits over each storage time (one year (1Y) and four years (4Y)) according to ageing technologies (Traditional: B; Alternative: O15, O30, O60, and N). For each analytical determination, different uppercase letters (A, B) in the same row denote significant differences between storage times (one year (1Y) and four years (4Y)) for each ageing modality by unpaired *t*-test (*p* < 0.05); different lowercase letters (a, b, c, d) in the same row denote significant differences between ageing modalities in each storage time by Tukey’s test (*p* < 0.05). LMW compounds: total LMWC—sum of content of individual LMW compounds; total phenolic acids—sum of content of phenolic acids; total furanic aldehydes—sum of content of furanic aldehydes; total phenolic aldehydes—sum of content of phenolic aldehydes.

**Table 2:** Effect of the storage time in the bottle on physicochemical characteristics of wine spirits aged by traditional and alternative technologies.

Analytical Parameters	1Y					4Y				
	B_1y_	O15_1y_	O30_1y_	O60_1y_	N_1y_	B_4y_	O15_4y_	O30_4y_	O60_4y_	N_4y_
Alcoholic strength by volume (% *v*/*v*)	76.38 ± 0.13 Aa	77.09 ± 0.11 Ab	77.09 ± 0.11 Ab	77.16 ± 0.17 Ab	76.72 ± 0.26 Aab	76.14 ± 0.20 Aa	77.05 ± 0.17 Ab	77.04 ± 0.21 Ab	77.24 ± 0.22 Ab	76.37 ± 0.33 Aa
Total acidity (g acetic acid/L AE)	0.89 ± 0.13 Bd	0.69 ± 0.01 Ac	0.66 ± 0.03 Ab	0.67 ± 0.01 Abc	0.58 ± 0.01 Aa	0.82 ± 0.03 Ad	0.74 ± 0.01 Bc	0.70 ± 0.01 Bb	0.71 ± 0.01 Bb	0.64 ± 0.02 Ba
Fixed acidity (g acetic acid/L AE)	0.44 ± 0.02 Bc	0.34 ± 0.01 Ab	0.32 ± 0.02 Ab	0.33 ± 0.01 Ab	0.27 ± 0.02 Aa	0.34 ± 0.02 Ac	0.33 ± 0.03 Abc	0.32 ± 0.02 Ab	0.32 ± 0.01 Abc	0.27 ± 0.01 Aa
Volatile acidity (g acetic acid/L AE)	0.45 ± 0.02 Ac	0.35 ± 0.01 Ab	0.35 ± 0.02 Ab	0.34 ± 0.01 Ab	0.31 ± 0.01 Aa	0.48 ± 0.03 Bc	0.41 ± 0.03 Bb	0.39 ± 0.01 Bba	0.39 ± 0.01 Bba	0.37 ± 0.03 Ba
Total dry extract (g/L)	2.37 ± 0.23 Ab	2.43 ± 0.08 Ab	2.27 ± 0.14 Ab	2.37 ± 0.04 Ab	2.03 ± 0.06 Aa	2.46 ± 0.10 Ac	2.44 ± 0.08 Abc	2.36 ± 0.01 Ab	2.38 ± 0.05 Abc	2.07 ± 0.01 Aa
pH	4.11 ± 0.05 Ba	4.16 ± 0.02 Bb	4.14 ± 0.01 Bab	4.18 ± 0.01 Bb	4.24 ± 0.02 Bc	3.97 ± 0.09 Aa	4.04 ± 0.04 Ab	4.07 ± 0.02 Ab	3.95 ± 0.03 Aa	4.12 ± 0.04 Ac

Results are expressed as mean values ± standard deviation (*n* = 4) of aged wine spirits over each storage time (one year (1Y) and four years (4Y)) according to ageing technologies (Traditional: B; Alternative: O15, O30, O60, and N). For each analytical determination, different uppercase letters (A, B) in the same row denote significant differences between storage times (one year (1Y) and four years (4Y)) for each ageing modality by unpaired *t*-test (*p* < 0.05); different lowercase letters (a, b, c, d) in the same row denote significant differences between ageing modalities in each storage time by Tukey’s test (*p* < 0.05).

**Table 3:** Characterisation of phenolic compounds in the wine spirits aged using the traditional and alternative technologies after four years of storage in the bottle by LC-DAD-ESI-MS/MS technique.

Peak	RT (min.)	λmax (nm)	Precursor Ion (***m***/***z***) [M − H]^+^	Precursor Ion (***m***/***z***) [M − H]^−^	Product Ions ***m***/***z*** (% Base Peak)	Tentative Identification	References
1	6.2			147	103 (100)	Citramalic acid	[88,89]
2	7.87			133	115 (100), 113 (40), 71 (20)	Malic acid	[88,90]
3	8.13	273		331	169 (100), 125 (35)	Galloyl glucose	[27,91,92]
4	8.42	271		331	169 (80), 271 (20), 211 (35), 125 (55)	Mono-*O*-galloyl-β-D-glucose	[27,92,93]
5	15.5	271		169	125 (100)	Gallic acid	[94,95,96]
6	20.03	284	127		127 (40), 109 (100), 81 (30)	5-Hydroxymethylfurfural	[97,98]
7	22.40	290, 326		153	153 (50), 109 (100)	Protocatechuic acid	[95,96,99]
8	29.85	281	97		97 (100), 69 (30)	Furfural	[97,98]
9	33.97	274		483	483 (100), 331 (20), 313 (30), 271 (20), 169 (60)	Di-*O*-galloyl-β-D-glucose 1	[27,91,95]
10	34.48	273		483	483 (100), 331 (20), 313 (30), 271 (20), 169 (60)	Di-*O*-galloyl-β-D-glucose 2	[27,91,95]
11	35.33	279		341	341 (100), 169 (10), 125 (10)	Gallic acid-glucoside	[27,92]
12	36.27	273		321	169 (100), 125 (10)	Digallate	[27,92]
13	39.95	273		635	635 (50), 483 (30), 465 (20), 313 (15), 211 (10), 169 (10)	Tri-*O*-galloyl-β-D-glucose	[27,95]
14	40.89	263, 292		167	167 (100), 152 (30), 108 (23), 123 (10)	Vanillic acid	[94,95]
15	41.86	273		197	197 (100), 161 (30), 182 (25), 153 (60)	Syringic acid	[94,95,96]
16	42.92	271		493	493 (100), 331 (10), 313 (10), 271 (20), 211 (30), 169 (10)	Monogalloyl-diglucose	[93,100]
17	44.05	278		289	289 (30), 245 (100), 203 (10), 179 (10)	Epicatechin	[101,102]
18	45.17	276		787	787 (30), 635 (20), 617 (20), 465 (15), 313 (10)	Tetra-*O*-galloyl-β-D-glucose	[27,95,103]
19	46.35	231, 325		193	193 (50), 178 (15), 149 (20), 134 (100),	Ferulic acid	[95,99]
20	46.65	276		939	939 (100), 787 (50), 769 (40), 635 (30), 617 (10)	Penta-*O*-galloyl-β-D-glucose	[27,92]
21	46.72	254, 365		301	301 (100), 229 (10)	Ellagic acid	[92,95,101]
22	47.25	280, 328		151	151 (60), 136 (100)	Vanillin	[94,95,104]
23	48.17	229, 306		181	181 (80), 166 (45), 151 (20)	Syringaldehyde	[95,96,101]
24	48.69	283, 307		167	167 (30), 109 (70)	Methyl protocatechuate	[94,98]
25	49.38	250, 361		585	585 (100), 301 (30)	Ellagic acid dimer dehydrated	[95,103,104]
26	50.48	320		307	307 (100), 261 (20), 235 (15)	3-Carbethoxymethyl-flavone	[94]
27	50.93	290		361	361 (40), 181 (50), 137 (100)	Homovanillic acid	[94]
28	51.66	271		663	663 (20), 331 (100), 169 (10)	Monogalloyl-glucose dimer	[91,103]
29	52.3	250, 362		433	443 (100), 301 (50)	Ellagic acid pentoside	[27,91,100]
30	52.85	244, 345		207	207 (100), 192 (50)	Sinapaldehyde	[95,103,104]
31	53.24	238, 340		177	177 (100), 162 (90)	Coniferaldehyde	[95,103,104]
33	54.02	274		197	197 (100), 169 (20), 125 (45)	Ethyl gallate	[94,105]
34	55.88	265, 362		447	447 (20), 285 (100)	Kaempherol-hexoside	[100]
35	57.82	265, 360		285	285 (100), 283 (40), 193 (50), 177 (20)	Kaempferol	[96,101]
36	59.28	254, 355		367	367 (100), 301 (80)	Ellagic acid derivative	[92,95]

## Data Availability

The data supporting the findings of this study are available within the article.
